# A Mobile App to Identify Lifestyle Indicators Related to Undergraduate Mental Health (Smart Healthy Campus): Observational App-Based Ecological Momentary Assessment

**DOI:** 10.2196/29160

**Published:** 2021-10-19

**Authors:** Chris Brogly, J Kevin Shoemaker, Daniel J Lizotte, Jacqueline K Kueper, Michael Bauer

**Affiliations:** 1 Faculty of Information and Media Studies Western University London, ON Canada; 2 Faculty of Health Sciences Western University London, ON Canada; 3 School of Kinesiology Western University London, ON Canada; 4 Department of Computer Science Western University London, ON Canada; 5 Department of Epidemiology and Biostatistics Western University London, ON Canada

**Keywords:** smartphones, undergraduates, mental health, lifestyle, postsecondary institutions, mHealth, mobile application, ecological momentary assessment, mobile phone

## Abstract

**Background:**

Undergraduate studies are challenging, and mental health issues can frequently occur in undergraduate students, straining campus resources that are already in demand for somatic problems. Cost-effective measures with ubiquitous devices, such as smartphones, offer the potential to deliver targeted interventions to monitor and affect lifestyle, which may result in improvements to student mental health. However, the avenues by which this can be done are not particularly well understood, especially in the Canadian context.

**Objective:**

The aim of this study is to deploy an initial version of the Smart Healthy Campus app at Western University, Canada, and to analyze corresponding data for associations between psychosocial factors (measured by a questionnaire) and behaviors associated with lifestyle (measured by smartphone sensors).

**Methods:**

This preliminary study was conducted as an observational app-based ecological momentary assessment. Undergraduate students were recruited over email, and sampling using a custom 7-item questionnaire occurred on a weekly basis.

**Results:**

First, the 7-item Smart Healthy Campus questionnaire, derived from fully validated questionnaires—such as the Brief Resilience Scale; General Anxiety Disorder-7; and Depression, Anxiety, and Stress Scale–21—was shown to significantly correlate with the mental health domains of these validated questionnaires, illustrating that it is a viable tool for a momentary assessment of an overview of undergraduate mental health. Second, data collected through the app were analyzed. There were 312 weekly responses and 813 sensor samples from 139 participants from March 2019 to March 2020; data collection concluded when COVID-19 was declared a pandemic. Demographic information was not collected in this preliminary study because of technical limitations. Approximately 69.8% (97/139) of participants only completed one survey, possibly because of the absence of any incentive. Given the limited amount of data, analysis was not conducted with respect to time, so all data were analyzed as a single collection. On the basis of mean rank, students showing more positive mental health through higher questionnaire scores tended to spend more time completing questionnaires, showed more signs of physical activity based on pedometers, and had their devices running less and plugged in charging less when sampled. In addition, based on mean rank, students on campus tended to report more positive mental health through higher questionnaire scores compared with those who were sampled off campus. Some data from students found in or near residences were also briefly examined.

**Conclusions:**

Given these limited data, participants tended to report a more positive overview of mental health when on campus and when showing signs of higher levels of physical activity. These early findings suggest that device sensors related to physical activity and location are useful for monitoring undergraduate students and designing interventions. However, much more sensor data are needed going forward, especially given the sweeping changes in undergraduate studies due to COVID-19.

## Introduction

Undergraduate study is demanding, and students often feel overwhelmed by their obligations [1]. Depression and anxiety frequently occur in undergraduate students [2], and these issues lead to decreased student experience [1] and strain on existing health resources [3]. Somatic health problems on their own are a major burden for universities [3]. Therefore, exploring cost-effective, preventative options or ways to mitigate mental health issues using ubiquitous resources is a sensible response. Undergraduates are highly connected [4] and frequently use mobile technology. Previous research has identified that mobile technology can be used in mental health because of the abundance of useful sensors in modern devices [5]. For undergraduate students, mobile technology is uniquely positioned to combine these valuable sensor data with self-reports using apps for new insights into mental health, as approaches for translating sensor data into behavioral markers relevant to mental health have been previously outlined [5,6]. Studies in this area have been previously conducted [6] but not necessarily using every available sensor and, to the best of the author’s knowledge, usually not at a Canadian university (although some do exist) [7]. Although Canadian universities can have similarities with universities in other countries (such as the United States), they are not identical, and there appears to be a general research gap for sensor-driven, smartphone-based mental health studies at Canadian universities. Similar studies conducted in other countries with comparable academic structures and settings can be relevant to Canadian undergraduates; however, they may not capture the nuanced experience of undergraduate education in Canada.

For the purposes of this research, it is important to clarify the term "mental health" as there are various definitions [8]. Here, the World Health Organization (WHO) definition is used, given the functional and practical requirements of an undergraduate study. Drawing upon the WHO definition for undergraduate study, it is essential that a student can "work productively" [9,10] and can "make a contribution" [9] to the university community. Students require the ability to "think" [9] for academic challenges and the ability to "emote or interact" [9], given that a university is ultimately an institution comprising people and not specific locations or buildings. Those who pursue undergraduate study essentially choose to do so as a step to "earn a living and enjoy life [9,11].

**Figure 1:**
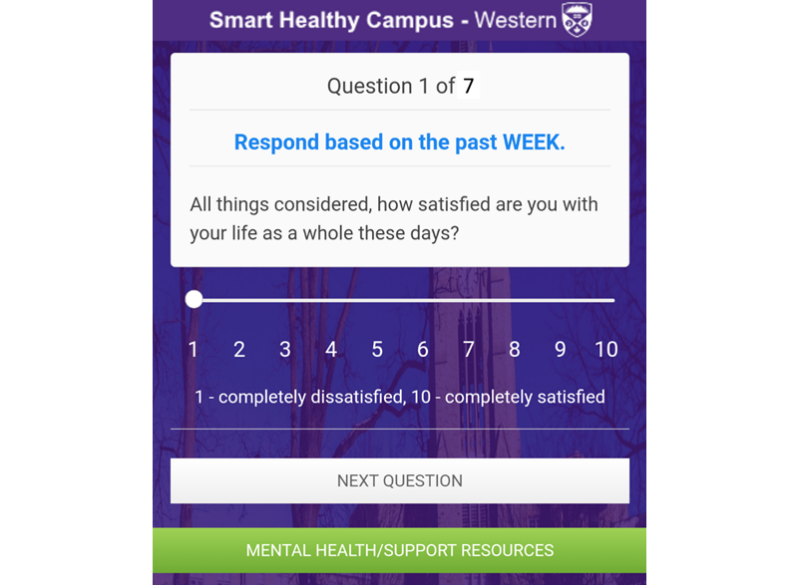
Question displayed by the Smart Healthy Campus app.

Given that Canadian universities [3] have recognized that undergraduate students are faced with stresses that challenge mental health, we developed the Smart Healthy Campus (SHC) app (Figure 1) to investigate the potential relationships between student lifestyle (measured by a short survey instrument) and relevant device sensors. The survey is a 7-item questionnaire that provides an overview of mental health and is included in Multimedia Appendix 1. The 7 questions were assembled from longer validated questionnaires [1]. The version of the app deployed at the time of writing contained a transaction-based sensor data collection system that captured readings relevant to aspects of lifestyle, which could be potential indicators of a student’s mental health.

The SHC app was deployed at Western University (formally known as the University of Western Ontario [UWO]) in London, Ontario, Canada, from March 2019 to March 2020 to all full-time undergraduate students. London’s population is about 383,000, and it is Canada’s 11th largest city. Western University is a relatively large public university in Ontario, Canada, with approximately 25,000 full-time undergraduates out of approximately 40,000 students in total as of 2020. The main campus is technically urban, although it is situated in a highly residential neighborhood north of London’s downtown core.

Participants were required to read the letter of information and consent at sign-up in the app. They then used their university email and self-identified as undergraduates and provided consent to this study, which was approved by the Health Sciences Research Ethics Board at Western University. Participants manually submitted questionnaire responses before any sensor readings were collected and sent to the study server.

The primary function of the SHC app is to facilitate an ecological momentary assessment (EMA). EMA is essentially used to observe and repeatedly sample study participants in their natural environments [12]. This preliminary iteration of the SHC study and app was sampled only on a weekly basis; however, the sample rate in subsequent work has been increased to a daily basis (see the Discussion section). The data collected comprised (1) the questionnaire results (providing a broad overview of mental health based on total score) and (2) sensor readings. The main goal of our work with this app is to determine if there were significant differences in the sensor readings between students who submitted a low-scoring response to the overall questionnaire compared with the sensor readings of those who submitted a high-scoring response. The reason for this interest is that, in the Canadian undergraduate context, sensors that might be the most useful with respect to understanding mental health have not been thoroughly investigated. If these are better understood, it would be considerably easier to build interventions aimed at evidence-based outcomes (including but not limited to notifications, suggestions, or app-based mentors or assistants) that might be included in new apps in this area. For instance, if there was evidence that Canadian students reporting a poor overview of mental health consistently displayed low readings from pedometers or minimal movement shown through GPS samples; interventions could attempt to alter lifestyle but then actually confirm a positive outcome by monitoring for increased activity in those sensors. We hope that this work will provide data to directly address this in the context of Canadian undergraduate students.

In addition, we investigated the relationship between distance to a university campus and sensor readings using GPS coordinates and geographic information system software. This was examined for 2 cases: (1) for samples that included questionnaire responses (providing a direct link to our focus on mental health) and (2) for samples that did not contain questionnaire responses (the app did not collect questionnaire responses for every consented transaction) as there were more samples in this case, and the data are still of interest.

## Methods

### Overview

This preliminary SHC study was conducted as a weekly EMA, which overall is a digitized, compacted version of most aspects of an in-person SHC pilot study [1]. The main advantage of moving to an app-based format was to make everything accessible to hundreds or potentially thousands of undergraduates rather than only a small classroom-sized group. However, although the in-person study focused on a mentorship intervention between upper- and lower-year students who hoped to improve mentors’ physical activity, resilience, and mental health, this digital SHC study omitted mentorship and simply observed participants as they progressed normally through their undergraduate study while using the SHC app. The idea being that SHC would still collect responses from participants regarding depression, anxiety, and resilience, but the extent to which these might occur would be connected to potential indicators coming from data from device sensors, such as pedometers or GPS, rather than from the impact of a mentorship intervention. In the pilot study, depression and anxiety were identified as having a major negative impact on undergraduates [1], and resilience was identified as the capacity to help cope with difficult life situations [1]. Having objective indicators of these items from device sensors could allow for new interventions such as notifications, suggestions, or app-based mentors or assistants targeting and monitoring those sensors. However, the questionnaires from the in-person study [1] for measuring these items would not fit well in an app format.

### Development of the SHC Questionnaire

Although short, validated mental health–related questionnaires were already available, we wanted to attempt to capture the same items as the original in-person SHC pilot study, albeit with a much smaller number of questions. It was not possible to do this with any existing questionnaires, as they did not cover all the domains of the original in-person SHC pilot study. The in-person SHC pilot study used several long-form validated questionnaires to measure general well-being, depression, anxiety, and resilience. These were issued using traditional survey methods [1]. Some examples of these instruments include the Brief Resilience Scale; Depression, Anxiety, and Stress Scale; and Mental Health Inventory. The problem with these items is that they might be difficult to complete in an app because of their combined length. As a result, a new SHC 7-item questionnaire (Multimedia Appendix 1) was created for SHC 1.0. All 7 questions were taken from the validated questionnaires with some minor edits to clarify that they are asking about the participant’s experience in the past week. These 7 questions would still provide similar measurements to the in-person SHC study, although at the expense of some accuracy, which was expected, given the significant reduction in the number of questions. Table 1 outlines the targeted areas of the questions and their scores. The total sum of the maximum scores for the 7 questions was 42. Table 2 outlines a tentative scoring system for the questionnaire, which was ultimately not used in this study. However, it is still related to how participant responses (and the corresponding sensor data) were grouped into the low- and high-scoring groups, as these were used for data analysis in the Results section.

In the Results section, we defined a low-scoring response as one with an SHC questionnaire total score ≤24 out of 42. We selected 24 as the low score cut-off, as it would include some marginal-to-acceptable total scores from 21 to 24 and all poor scores. We defined a high-scoring response as one with an SHC questionnaire total score >24, as it is difficult to obtain a full score of 42/42 in many cases. For instance, the last question asks about the days with significant exercise in the last week, and each day counts as 1 point, hence the seemingly marginal standard for a high-scoring response. This was done because of the limited amount of data for analysis.

**Table 1:** Coverage of the Smart Healthy Campus mental health overview questionnaire and scoring.

Question number^a^	Behavioral marker	Points	Question source
1	Life satisfaction	10	[13]
2	Psychological well-being	6	[14]
3	Resilience	5	[15]
4	Anxiety	4	[16]
5	Depression	4	[17]
6	Community connectedness	6	[18]
7	Physical activity	7	[19]

^a^Questionnaire score total: 42 points.

**Table 2:** Smart Healthy Campus 1.0 7-item questionnaire tentative scoring system.

Range	Tentative rating (disused in this work but relevant to the following column)	Assigned to a low-scoring or high-scoring group for data analysis in this work
0-6	Very poor	Low
7-13	Poor	Low
14-21	Marginal	Low
22-27	Acceptable	Low (score ≤24); high (score >24)
28-35	Good	High
36-42	Very good	High

### Development of the SHC App

#### App Requirements

There were 3 key app requirements for SHC. The first key requirement of the app was to administer the 7-item SHC questionnaire and collect responses. Sliders and multiple-choice options were used depending on the question. The 7-item questionnaire incorporated into this study was asked on a weekly basis by the app.

The second key requirement was the ability to collect data from various device sensors. Although the focus is almost exclusively on smartphones for this work, we still use the term "device sensor" as tablets, iPads (Apple Inc), or iPods (Apple Inc) were also able to run the app (although no one used them to participate in this study). Data collection from device sensors was required to occur for any of 3 important events: when a participant requested to complete a weekly survey (a Request event), when a participant submitted responses to a survey (a Response event), or when a participant used a “Mental Health/Support Resources” panel (a HelpNow event).

The third key requirement was to make it easy for students to find information in a single place about the support services that were available to them. The app would contain a Mental Health Support Resources button, which opened a panel that provided participants with easy access to key crisis services if they felt that they needed them. This included phone numbers, website links, and general information about the types of support resources. This requirement was derived from the finding of one assessment that facilitating access to crisis support had evidence for suicide prevention [20]. If a participant pressed the Mental Health/Support Resources button, they were to be presented with a panel that asked them if they would like to consent to data collection about how they used the Mental Health/Support Resources panel. If consent was given, then data were to be sent to and recorded by the server on what parts of the panel were used, such as if they expressed interest in a service in the app or went further and selected a website link or a phone number. Combined with questionnaire and sensor data, these data are important, as they may provide markers that indicate that a student needs additional support. In addition, device sensor data were collected during this event.

#### Design and Architecture

We refer to the underlying client-server software for the SHC app as EMAX1 (EMA Extensions 1st edition software). The general idea behind EMAX1 is that it will eventually become a generic app for EMA-type studies instead of having a specific focus or name such as Smart Healthy Campus. Instead, participants will simply download a single app and select the survey they want to participate in; surveys will be able to adjust sensor data collection to their specific needs.

The EMAX1-based SHC software was designed using a standard client-server architecture. Participants used the EMAX1 client app to communicate with the EMAX1 server. The server software runs in the cloud and responds to any requests that are generated by clients over hypertext transfer protocol secure. In general, for SHC, which is the only EMAX1-based app, communication is minimized to improve responsiveness over potentially poor data connections. For instance, the survey questions were originally sent from the server to the app to allow for easy reconfiguration; however, eventually, as much as possible was moved into the client to maximize responsiveness at the expense of some flexibility.

#### Implementation

The EMAX1 client app was developed using Apache Cordova. Cordova apps are implemented as webpages and can be built into apps for Android and iOS. Cordova was used as Android and iOS share the same HTML, Cascading Style Sheets, or JavaScript codebase, which reduced development efforts. However, even with a shared codebase, some conditions needed to be in place to check system variables for detecting which operating system was running to correctly obtain hardware sensor data, because of differences between iOS and Android. The EMAX1 server software used for SHC was implemented in Python. The server is not described in detail in this paper, as the Python implementation relies on standard libraries and currently existing technology.

### Recruitment and Consent

Recruitment was conducted at Western University primarily from March to December 2019. Web links to connect undergraduate students to the SHC app were distributed to certain undergraduate classes and sent to relevant student wellness stakeholders in undergraduate faculties. In-class recruitment sessions were held in high-enrollment classes (≥150 students) where professors allowed them. Mass email recruitment to all undergraduates was also conducted. Participants were able to join the SHC at any time by downloading the app and registering to participate using their university (UWO) email account. Students were required to read the letter of information and had the option of consenting to express their interest in connecting their records from student health services and student recreation services to the SHC study. Unfortunately, connecting to the records was ultimately not possible in this preliminary study because of logistic issues, although subsequent studies are expected to specifically connect to campus health records.

The SHC app solicited responses via push notifications (small popup messages on the participant’s device) to existing users, usually 2 times per week: once on Tuesday in the afternoon and then again between Friday and Sunday as a final reminder. At least one previous study focused on the timing and frequency of push notifications for use in an app and suggested that static notifications delivered at a recurring time are acceptable [21]. Notification emails were also occasionally sent to registered participants at the start of each weekly survey on Monday. Occasionally, if the app experienced technical issues, additional push notifications or emails were sent to participants to let them know the system was running again.

### Analysis

All data were analyzed as a single collection, and there was no analysis with respect to time because of the limited amount of data. In addition, as Shapiro-Wilk tests showed that data were not normally distributed, we relied on nonparametric statistical tests that compare the mean ranks of the groups. The Mann-Whitney U test was used to compare the mean ranks of the 2 groups, and the Kruskal-Wallis test with a post hoc Wilcoxon rank-sum test with Bonferroni correction was used to compare the mean ranks of 3 or more groups. No corrective action was required for the missing data. For example, during a sensor sample, if we did not obtain a GPS coordinate but did obtain the battery level, then we would only include any associated data related to the battery level for statistical tests, such as a questionnaire score. Data analysis was conducted using a combination of R 3.4.4 and SPSS 26 for Linux.

## Results

### Overview

**Figure 2:**
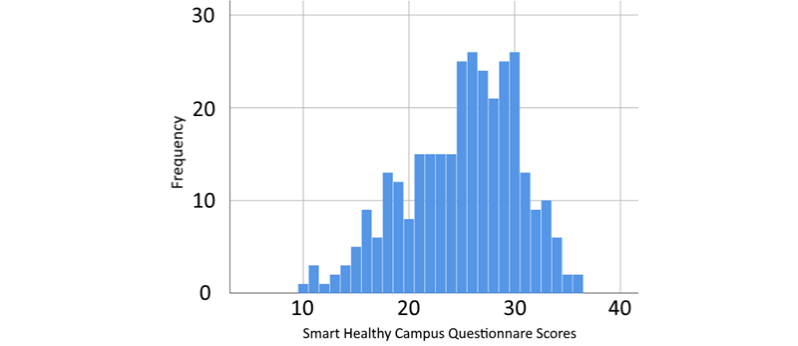
Histogram plot of all participants’ Smart Healthy Campus questionnaire scores. The maximum score is 42 (mean 25.08, SD 5.32; N=312).

Data collection for this preliminary work occurred from March 2019 to March 2020 and ended around the time the WHO declared COVID-19 a pandemic. There was no specific sample size for this work; however, it was hoped that eventually, approximately 100-300 students would participate on a weekly basis (this would happen for our revised COVID-19 pandemic app called Student Pandemic Experience [SPE] for some time). For SHC 1.0, this did not turn out to be a feasible goal (possibly because of the lack of any incentive system), and enrollment and participation fell well short of this. Owing to technical limitations in both the study and the app, both being in the early stages of this work, demographic information was not collected. This omission limits the grouping variables for the analysis of the results. For instance, we did not examine any subgroups. This was later addressed after SHC 1.0 concluded to allow for demographic data collection in future studies (see the Discussion section). A summary of collected data is presented below in Table 3. Figure 2 is a histogram of all response scores.

**Table 3:** Summary of collected data.

Item and description				Values, n (%)
**Participants (the total number of participants who enrolled in the study)**				139 (100)
	iOS users	How many participants used an Apple device?	121 (87.1)	
	Android users	How many participants used an Android device?	18 (12.9)	
	Participants responding for >1 week	The number of participants that completed more than one weekly survey.	42 (30.2)	
**Total number of samples (Response+Request+HelpNow event counts)**				813 (100)
	Response events	The number of questionnaire responses. Response events consist of a questionnaire response and data from all sensors at the time they occurred.	312 (38.4)	
	Request events	The number of times participants tried to obtain a questionnaire. Request events only contain data from all sensors at the time they occurred.	492 (60.5)	
	HelpNow events	The number of times participants used the mental health resources panel and consented to its data collection features.	9 (1.1)	

In the first subsection, we show that the SHC questionnaire does have significant correlations with the selected mental health domains. Then, an analysis of the preliminary data collected is presented from different perspectives in the following 3 subsections. For the first 2 sections, focus is placed on how the participant responses to the app questionnaire (the self-reported mental health overview) related to the readings collected from device sensors (more objective indicators of lifestyle). These data came only from Response events (when participants submitted answers to a questionnaire). The third and final analysis was based on any sensor samples containing GPS data, which came from both Request (only obtaining the weekly questionnaire) and Response (responding to the weekly questionnaire) events from participants. These were combined for analysis, as including the readings from Request events increased the value of N for statistical tests based on geographic information for on- or off-campus locations. No analysis of the HelpNow events could be presented here because of a lack of data.

The results of the analyses shown below provide some limited data as to what sensors would be most relevant for monitoring or designing interventions (ie, targeted suggestions, notifications, or app-based mentors or assistants) for undergraduate mental health.

### Correlations Between the SHC Questionnaire and Mental Health Domains

Tests for correlations were conducted between the SHC app questionnaire and domains for resilience, anxiety, and depression. These domains comprised individual questions taken from fully validated questionnaires used in the original SHC course-based study [1]. Individual questions were selected from established questionnaires using a data-driven method (devised but not yet published by authors; Kueper and Lizotte, unpublished data, April 2019) applied to completed questionnaire data from a similar population, as well as undergraduate students at the same university who were participants in the original in-person SHC study. The method essentially ranks questions based on explained variability and was used to select a subset of the most highly ranked questions to cover each of the target measurement domains. When questions were similarly ranked, the one that would require minimal modification for quick administration using an app was selected. Where needed, questions included in the app were modified in terms of the period for participants to consider but not in terms of major word content. These domains are shown in Textbox 1.

**Domains and questions for correlation tests.**

**Resilience**

- Entire Brief Resilience Scale questionnaire

**Anxiety**

- Entire General Anxiety Disorder-7 questionnaire
- Depression, Anxiety, and Stress Scale (DASS): 1, 3, 8, 10, 15, 19, 20
- Mental Health Inventory (MHI): 4, 10, 11, 18
- One Visual Analog Scale–type question

**Depression**

- DASS: 2, 5, 9,12, 14, 17, 21
- MHI: 2, 9, 12, 14

**Other**

- DASS: 4, 6, 7, 11, 13, 16, 18
- MHI: 1, 3, 7, 8, 13 15, 16, 17

For correlation tests, we compared the results of 1 week of in-class responses to the questions covering the 4 domains with those of responses to relevant parts of the app questionnaire reduction. The Spearman rank correlation test was used as the main test statistic (although Pearson correlation tests were run anyway and results were very similar; they are not included here). The results are shown in Table 4.

**Table 4:** Results of Spearman correlation between in-class responses and app questionnaire reduction responses.

Correlation	Domains	Sample, n	Spearman ρ (SE)	*P* value
Sum of questions 1-5	Resilience, anxiety, depression, and other	48	0.39 (0.13)	.005
Life satisfaction question	Resilience, depression, and anxiety	48	−0.23 (0.14)	.11
Resilience question	Resilience	55	−0.62^a^ (0.10)	<.001
Depression question	Depression	52^b^	0.52 (0.10)	<.001
Anxiety question	Anxiety	50^b^	0.71 (0.07)	<.001

^a^Question coding was reversed during this test, which resulted in a negative correlation.

^b^n is different for some tests, which shows that some participants only completed certain full surveys in class.

On the basis of these results, the Spearman rank correlation between the selected items that were chosen for the app at the time of writing was generally moderate (Spearman ρ=0.39; *P*<.001) to strong (Spearman ρ=0.71; *P*<.001). An exception was app question 1 from the World Values Survey 2012, which turned out not to have a statistically significant correlation with the resilience, depression, and anxiety domains, although the focus was not quite the same.

Overall, the results showed that the 7-item SHC questionnaire implemented in the app had a useful correlation with the full mental health surveys, whereas the single question measures for resilience, depression, and anxiety had strong correlations with their respective domains from the full surveys. Some loss of precision was expected, given that the app questionnaire was very short. We argue that some loss of precision is acceptable for this type of observational research as the advantage is that the questionnaire reduction can be completed in far less time than the full surveys, making it appropriate for use in the app.

### Difference Between On-Campus and Off-Campus Questionnaire Response Scores

The first analysis of interest was to determine, using GPS data, if there were any differences in the questionnaire scores between samples from students that were responding to weekly surveys on campus compared with samples from those that were responding to the surveys off campus. This was examined, as living off campus can help contribute to a decrease in daily physical activity during the undergraduate study [22]. To do this, all Response event records with identifiable GPS information (some records did not have GPS information because of transmission issues, or participants denied the request to access GPS) were used. On-campus students were within 1.5 km of coordinates at the center of the main campus of UWO. Everyone else was classified as an off-campus student. A Mann-Whitney U test was conducted between these 2 groups to determine if there were any significant differences (on campus, mean rank=121.41; off campus, mean rank=89.05; Mann-Whitney U=3914.5; *P*<.001).

The results of the test showed that there was a significant difference between the 2 groups (*P*<.001) and that the on-campus students actually had a higher mean rank than those off campus, which suggested, at least based on these samples, that students on campus actually reported a more positive overview of mental health than those who were off campus.

### Difference Between Sensor Readings From Low-Scoring Responses Compared With High-Scoring Responses

The second analysis was conducted to determine if there were significant differences in the sensor readings from those submitting low responses to weekly questionnaires compared with those who submitted higher responses. Differences in sensor readings were tested from 2 perspectives: one without GPS data used as a grouping variable and one with GPS to create on- and off-campus groups.

We defined a low-scoring response as one with an SHC questionnaire total score ≤24 out of 42. We selected 24 as the low score cut-off, as it would include some marginal total scores from 21 to 24 and the poor (≤50%) scores below that. We defined a high-scoring response as one with an SHC questionnaire total score >24, as it is difficult to obtain a full score of 42/42 in many cases. For instance, the last question asks about the days with significant exercise in the last week, and each day counts as 1 point, hence the seemingly marginal standard for a high-scoring response.

### Differences Between Low-Scoring and High-Scoring Responses—No Additional GPS Grouping

The first perspective was simply based on total questionnaire scores, where a low-scoring response group and a high-scoring response group were tested for any significant differences in sensor data using a Mann-Whitney U test. The results are shown in Table 5.

The results of the Mann-Whitney U tests in Table 5 yielded some significant findings with the sensors. The mean ranks of these items are listed in Table 6.

Participants with low-scoring responses, based on mean rank, tended to be plugged in (charging) more, had higher uptimes (possibly as a result of charging), showed less physical activity based on pedometer readings, used the app less, and took less time to complete the weekly responses.

Conversely, participants with high-scoring responses, based on mean rank, were plugged in less and had their devices turned on less. However, they spent more time using the app and responding to the weekly surveys. They also showed greater levels of physical activity based on the pedometer readings.

**Table 5:** Mann-Whitney U tests between low- and high-scoring samples for significant differences between sensor readings.

Sensor item	New use?	Low-scoring samples^a^	High-scoring samples^b^	Mann-Whitney U	*P* value^c^
Plugged-in flag	No	55	83	1477.5	<.001
Battery level	No	64	135	4074.5	.51
User CPU^d^ time	Yes^e^	87	169	6916.0	.44
Idle CPU time	Yes	87	169	6818.0	.34
Total CPU time	Yes	87	169	6897.0	.42
User CPU percentage	Yes	80	153	5515.0	.22
User idle percentage	Yes	80	153	5537.0	.23
Available RAM	Yes	87	169	6386.0	.09
System uptime	No	121	189	9557.0	.006
Uptime with sleep	No	121	189	9524.0	.005
Pedometer step count	No	73	58	1564.0	.01
Pedometer distance	No	77	61	1738.0	.009
Pedometer floors up	No	77	61	1600.5	.001
Pedometer floors down	No	77	61	1617.5	.002
Pedometer 2 step count	No	13	10	51.0	.23
App use time	Yes	123	189	9082.0	.001
Survey time to complete	Yes	123	189	9678.0	.01
Resource panel time	Yes	123	189	11591.0	.76

^a^Total Smart Healthy Campus questionnaire score ≤ 24.

^b^Total Smart Healthy Campus questionnaire score >24.

^c^*P*<.01 is considered significant.

^d^CPU: central processing unit.

^e^Anything marked with “Yes” was, to the best of the author’s knowledge in 2020, a new use of this sensor for this type of work.

**Table 6:** Mean ranks for sensor items with significant differences, low versus high questionnaire response scores.

Significant sensor item	Low-scoring questionnaire response scores, mean rank	High-scoring questionnaire response scores, mean rank
Plugged-in flag	84.14	59.80
System uptime	171.02	145.57
System uptime with sleep	171.29	145.39
Pedometer step count^a^	58.42^a^	75.53^a^
Pedometer distance	61.57	79.51
Pedometer floors up	59.79	81.76
Pedometer floors down	60.01	81.48
App use time	135.84	169.95
Survey time to complete^a^	140.68^a^	166.79^a^

^a^These items had *P* values very close to the level considered significant (*P*=.01), suggesting that tests with more data may report direct significance.

### Differences Between Low-Scoring and High-Scoring Responses With On- or Off-Campus GPS Grouping

The second perspective was based on both total questionnaire scores and GPS data, resulting in 4 groups: low-scoring on-campus responses, high-scoring on-campus responses, low-scoring off-campus responses, and high-scoring off-campus responses. On-campus students were within 1.5 km of coordinates at the center of the main campus of UWO. Testing for any significant differences in sensor data among any of these 4 groups was completed using a Kruskal-Wallis test. These results are shown in Table 7, with the mean ranks from the Wilcoxon post hoc test in Table 8.

**Table 7:** Kruskal-Wallis tests for significant differences between sensor readings, including low- and high-scoring samples for both on- and off-campus students.

Sensor	New use?	Sample, n						Chi-square (*df*)		*P* value
		On campus			Off campus					
		Low scoring	High scoring	Low scoring		High scoring				
Plugged-in flag	No	14	53	28		14	32.3 (3)		<.001	
Battery level	No	20	91	28		18	3.9 (3)		.27	
User CPU^a^ time	Yes^b^	34	103	44		48	0.9 (3)		.82	
Idle CPU time	Yes	34	103	44		48	7.3 (3)		.06	
Total CPU time	Yes	34	103	44		48	4.5 (3)		.22	
User CPU percentage	Yes	34	94	37		42	18.1 (3)		<.001	
User idle percentage	Yes	34	94	37		42	17.7 (3)		<.001	
Available RAM	Yes	34	103	44		48	7.0 (3)		.07	
System uptime	No	34	103	44		48	63.7 (3)		<.001	
Uptime with sleep	No	34	103	44		48	68.9 (3)		<.001	
Pedometer step count	No	19	20	28		34	1.9 (3)		.59	
Pedometer distance	No	22	23	28		34	0.7 (3)		.88	
Pedometer floors up	No	22	23	28		34	2.9 (3)		.41	
Pedometer floors down	No	22	23	28		34	2.1 (3)		.55	
Pedometer 2 step count	No	N/A^c^	N/A	13		N/A	2.2 (3)		.33	
App use time	Yes	34	103	44		48	42.3 (3)		.001	
Survey time to complete	Yes	34	103	44		48	30.4 (3)		.001	
Resource panel time	Yes	34	103	44		48	1.2 (3)		.75	

^a^CPU: central processing unit.

^b^Anything marked with “Yes” was, to the best of the author’s knowledge in 2020, a new use of this sensor for this type of work.

^c^N/A: not applicable.

**Table 8:** Mean ranks for sensor items with significant differences, low-scoring versus high-scoring questionnaire response scores, on campus and off campus.

Significant sensor item	Mean rank				
	On campus			Off campus	
	Low-scoring	High-scoring	Low-scoring		High-scoring
Plugged in	48.71	41.79	77.36		66.57
User CPU^a^ percentage	86.44	98.71	140.76		97.67
User idle percentage	121.74	109.13	67.68		110.17
Uptime	117.32	80.78	156.07		149.14
Uptime with sleep	116.54	79.46	156.95		151.71
App use time	112.79	143.72	94.66		73.58
Survey time to complete	95.91	138.65	113.98		78.71

^a^CPU: central processing unit.

Although the results shown in Tables 7 and 8 are limited, given the small values of N, they suggest that sensor values can be interpreted depending on various contexts, given our experimentation with GPS coordinates here. In this paper, context is essentially the grouping variable used. For instance, the questionnaire score was always intended to be used to contextualize the data for this initial study as we compare low scores with high scores here. However, GPS location also appears to provide some useful context, given that we found some significant differences among the 4 groups, shown initially in Table 7 through significant *P* values and then in Table 8 with mean ranks from the Wilcoxon post hoc test.

The results of the Kruskal-Wallis tests, using GPS as an on- or off-campus grouping variable, showed some significant findings. The mean ranks of these items are listed in Table 8.

Pairwise comparisons using the Wilcoxon rank-sum test with Bonferroni correction were conducted on the 4 groups shown in Table 5 to determine specific differences, the significance of which is shown by any mention of *P* values in the remainder of this section.

For the plugged-in sensor, there was a significant difference between the high-scoring on-campus samples and low-scoring off-campus samples (*P*<.001), and high-scoring off-campus samples (*P*=.008). This suggests that more participants with higher scores were not plugged in (charging or charged) on campus. This may suggest more movement on campus.

User central processing unit (CPU) time is consumed by the apps launched by the user. User CPU time percentage and user CPU idle time percentage, in general, have an inverse relationship. User CPU time, based on mean ranks from 3 pairwise comparisons, tended to be higher for low-scoring off-campus samples (*P*<.001; *P*=.003; *P*=.004) compared with any on-campus samples and high-scoring off-campus samples. This finding suggests that low-scoring off-campus participants put more computational load from user software on their devices, suggesting higher general use.

Low-scoring on-campus samples (*P*=.004), low-scoring off-campus samples (*P*<.001), and high-scoring off-campus samples (*P*<.001) tended to have significantly higher uptimes (in milliseconds) based on mean rank compared with high-scoring on-campus samples. Similar results were found for uptime with sleep (when the device is left on but is not used for a significant amount of time, such as overnight). This suggests that high-scoring on-campus students use devices less.

For app use time, high-scoring on-campus samples tended to have, based on their mean ranks, significantly higher SHC app use time than low-scoring off-campus and high-scoring off-campus samples but not low-scoring on-campus samples. This result somewhat contradicts the general test between low- and high-scoring questionnaire scores in Tables 7 and 8, where the low-scoring group had, based on mean rank, less app use time.

For survey time, there was only a significant difference between on-campus high-scoring samples and off-campus high-scoring samples (*P*<.001), where the on-campus high-scoring samples tended to report longer survey completion times.

### Differences Among On-Campus, Limited In-Residence, and Off-Campus Sensor Data Using All Samples, No Questionnaire Scores

The last analysis was completed on the remaining preliminary data using additional Request events, where on- or off-campus GPS data were available but questionnaire response scores were not. The Request samples were combined with the Response sample sensor data to increase the value of N for the statistical tests. These results are suspected to be less reliable than those from the previous 2 subsections, given the amalgamation of the events.

First, Wilcoxon rank-sum tests were conducted between the Request and Response event groups for each sensor item to determine if there was a statistically significant difference between the 2 types of requests. If there was a significant difference between the Request and Response events for a sensor item, it was not analyzed in this section. For instance, app use time will always be significantly lower in a Request compared with a Response, as time will have passed in the Response event. The results for the acceptable sensor items are shown in Table 9.

**Table 9:** Mean ranks for sensor items with significant differences, on-campus versus residence versus off-campus locations.

Significant sensor item^a^	On-campus			Residence			Off-campus	
	Mean rank	Sample, n	Mean rank		Sample, n	Mean rank		Sample, n
Battery level	177.45	240	96.28		16	202.02		105
User time	211.38	166	323.51		35	213.66		241
Idle time	215.84	166	367.09		35	204.26		241
Total time	213.06	166	363.54		35	206.68		241
Uptime	202.88	280	364.36		47	379.41		260
Uptime with sleep	199.59	280	353.03		47	385.00		260

^a^*P*<.01.

Pairwise comparisons using the Wilcoxon rank-sum test with Bonferroni correction were conducted on the 3 groups shown in Table 6 to determine specific differences.

For battery level, there was a significant difference between the on-campus and residence samples (*P*=.008). On the basis of mean rank, on-campus samples tended to have higher battery levels than those from residence samples (possibly as participants returned to their residence with drained batteries). There was also a significant difference between samples from residence compared with samples from off campus (*P*<.001), with off-campus samples reporting, based on their mean ranks, higher charge levels. This suggests that more participants may have been reporting in with access to a charger, likely at home.

For user CPU time, there was a significant difference between the on-campus and residence samples (*P*=.008) and residence and off-campus samples (*P*<.001), with higher user CPU times tending to be from residence samples based on their mean ranks. The same results were found for idle CPU time (*P*<.001; *P*<.001) and total CPU time (*P*<.001; *P*<.001). These limited results may suggest that participants in residences are running their phones longer and actively using them more. However, this finding may be less reliable than previous ones in terms of CPU time because of the amalgamation of the Request and Response sensor data.

## Discussion

### Limitations

Overall, the results presented here are preliminary, given the limited amount of data. As this was exploratory observational research, the effects of confounding factors could not be controlled. In addition, we relied on self-identification of undergraduate status because of privacy issues related to the release of information from the university registrar. However, sign-ups largely coincided with recruitment from undergraduate classes and mass emails to undergraduates. In general, personal smartphones are commonly used by undergraduates at Western University; however, it is possible that some students may not have permanent access to one for various reasons, such as losing a device or perhaps for financial reasons, which may affect participation in any smartphone-based study from time to time and as such is always a possible limitation.

Other limitations were that the number of samples (n=813), total number of participants (n=139), and retention to a second weekly sampling (42 participants) were low, given that this study was accessible to a university campus over nearly a year. This stemmed primarily from incremental revisions to the study, resulting in limited recruitment, system downtime for maintenance or other issues, and possibly from limited interest as no incentives were available or offered to participants over this period. Owing to technical limitations with the study and the SHC app, it was not possible to collect information on why participation was low or if participants uninstalled the app. In addition, we did not take differences in the undergraduate year of study, or program, sex, or any other demographic items into account for this preliminary analysis. In part, this was because we expected to be able to obtain this from the university; we now ask for it directly in the new SHC 2.0 and SPE apps. Another issue was that there was no continuous or longitudinal analysis of individual responses over time; it was not feasible to perform this, given that retention rates were low. It is expected with version 2.0 of the app and intensified recruitment efforts that a longitudinal analysis may become possible.

It is also important to note that the results may not necessarily be representative of all institutions, as Western University is a relatively large (approximately 40,000 total enrollments) publicly funded university with an urban campus situated in a heavily residential area. In addition, these data were collected before the shift to web-based learning at Western University, resulting from COVID-19. It is important to note that going forward, numerous measures resulting from COVID-19 (even as vaccination rates increase in Canada) are likely to affect Western University in 2021 and in some time beyond. As a result, the limited data from this study will not be representative of conditions for some time, given the massive impact of COVID-19 on universities.

### Impact

This work begins with the process of establishing an evidence base for future digital interventions for the Canadian undergraduate population. It is the first study in a line of research to identify associations between psychosocial factors (captured by our SHC questionnaire) and behaviors (measured by smartphone sensors) in undergraduates at a Canadian university. When the associations between psychosocial factors and behaviors are known, evidence-based, cost-effective digital interventions, such as notifications and chatbots, can be developed to intervene in the everyday experiences of students. These offer the potential to alter lifestyle patterns to align with behaviors related to positive mental health. As app-based interventions can be widely distributed, they are expected to be used to reduce the extent of mental health concerns and burdens on campus health resources. Overall, in this initial study, we found that on-campus location and higher levels of physical activity were associated with more positive mental health. Similar studies on undergraduates from the United States and China have found generally comparable results [23,24].

### Next Steps

These initial results regarding sensor indicators are promising but limited, and it is apparent that increased data collection would yield more meaningful results. As a result, the underlying EMAX1 client or server software has been upgraded to EMAX2 (EMA Extensions 2nd edition software) and is supporting a newer SHC 2.0 app, which includes a number of general improvements, such as increasing the number of sensors used for data collection, background data sampling, daily sampling, and the addition of a points-based incentive system. The SHC 2.0 app also includes a more robust sign-up process that addresses differences in enrollment status and some demographic information, items that were not always feasible to obtain another way, such as from the registrar. The SPE app to study COVID-19 was also built on EMAX2 and is available on iOS and Android at the time of writing. It is expected that the SHC and SPE EMAX2–based research apps will eventually be replaced by a single unified EMAX3 (EMA Extensions 3rd edition software) app for iOS and Android, which will include a full configuration system to conduct additional EMA-based studies.

### Conclusions

Although previous studies have been conducted in this area, data directly from Canadian undergraduates are limited, impeding the development of evidence-based mental health interventions using the capabilities of readily accessible smartphones. In this work, an initial attempt was made to address this in the context of Canadian undergraduates, where mental health remains an issue of concern for both students and administrators.

## Appendix

Multimedia Appendix 1 Smart Healthy Campus questionnaire.

## Abbreviations

CPU: central processing unit
EMA: ecological momentary assessment
EMAX1: Ecological Momentary Assessment Extensions 1st edition software
EMAX2: Ecological Momentary Assessment Extensions 2nd edition software
EMAX3: Ecological Momentary Assessment Extensions 3rd edition software
SHC: Smart Healthy Campus
SPE: Student Pandemic Experience
UWO: University of Western Ontario (Western University, Canada)
WHO: World Health Organization
