# Supplementary material for: A Mobile App to Identify Lifestyle Indicators Related to Undergraduate Mental Health (Smart Healthy Campus): Observational App-Based Ecological Momentary Assessment
Source: JMIR Form Res. 2021 Oct 19;5(10):e29160. doi: 10.2196/29160 (PMC8564659; doi:10.2196/29160)
Supplement: Multimedia Appendix 1 [file formative_v5i10e29160_app1.docx]

Bold font is what was shown on the app to respondents (both question and response options). Response option numerical values in brackets are required for scoring. The source for each question is included.

Questions:

1. **All things considered, how satisfied are you with your life as a whole these days?**

**Daily variant: All things considered, how satisfied are you with your life today?**

Response options: **range from 1 to 10 where 1 means you are “completely dissatisfied” and 10 means you are “completely satisfied”.**

(Source:World Values Survey 2012 (<http://www.worldvaluessurvey.org/WVSDocumentationWV6.jsp>)

1. **Over the past week, how much of the time have you felt cheerful, light-hearted?**

**Daily Variant: How much of the time have you felt cheerful, light-hearted today?**

Response options: (6) **None of the time,** (5) **A little bit of the time**, (4) **Some of the time**, (3) **A good bit of the time**, (2) **Most of the time**, (1) **All of the time.**

(Modified MHI Question 13)

1. **Please indicate how much you agree with the following statement: It is hard for me to snap back when something bad happens.**

**Daily Variant: Please indicate how much you agree with the following statement: It was hard for me to snap back when something bad happened today.**

Response options: (1) **Strongly disagree,** (2) **Disagree,** (3) **Neutral,** (4) **Agree,** (5) **Strongly Agree.**

(Modified BRS Question 4)

1. **Over the past week, how often have you been bothered by not being able to stop or control worrying?**

**Daily Variant: How often have you been bothered by not being able to stop or control worrying today?**

Response options: (1) **Not at all**, (2) **Several days**, (3) **More than half the days**, (4) **Nearly every day**.

(Modified GAD-7 Question 2)

1. **Please indicate how much the following statement applied to you over the past week: I felt I wasn’t worth much as a person.**

**Daily Variant: Please indicate how much the following statement applied to you today: I felt I wasn’t worth much as a person.**

Response options: (1) **Did not apply to me at all**, (2) **Applied to me to some degree, or some of the time**, (3) **Applied to me a considerable degree or a good part of the time,** (4) **Applied to me much or most of the time.**

(Modified DASS-21 Question 17 (taken from https://maic.qld.gov.au/wp-content/uploads/2016/07/DASS-21.pdf)

1. **Include in app the header text (‘Circle….’) with each circle representation as a response option. Figure title not required.**

**Daily Variant: None**


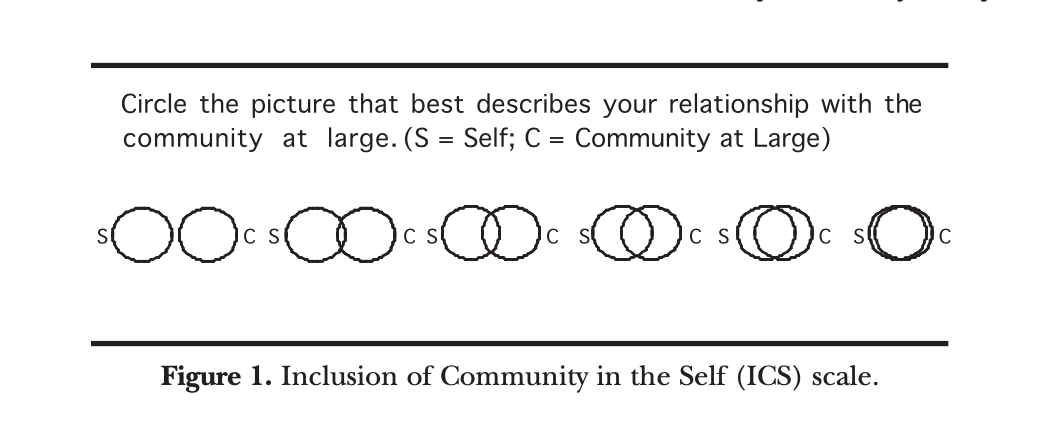


(Source: Inclusion of community in self scale: A single-item pictorial measure of community connectedness)

Response values for this question will be numeric on a scale of 1 (least inclusion) to 6 (most inclusion).

1. **In the past week, on how many days have you done a total of 30 minutes or more of physical activity, which was enough to raise your breathing rate. This may include sport, exercise, and brisk walking or cycling for recreation or to get to and from places, but should not include housework or physical activity that may be part of a job you have outside school.**

**Daily Variant: None**

Response options: range from **0 to 7 days**.

(Source: Milton Single Item Physical Activity Measure.) <https://pdfs.semanticscholar.org/f70f/3615a54718b1c49b0db388656ac079310a43.pdf>
